# Attitudes of Young Tri-City Residents toward Game Meat in the Context of Food Neophobia and a Tendency to Look for Diversity in Food

**DOI:** 10.3390/ijerph20053815

**Published:** 2023-02-21

**Authors:** Dominika Mesinger, Aneta Ocieczek, Witold Kozirok, Tomasz Owczarek

**Affiliations:** Faculty of Management and Quality Science, Gdynia Maritime University, 81-225 Gdynia, Poland

**Keywords:** game meat, attitudes, FNS, VARSEEK, GMAS, sustainable consumption, food

## Abstract

To conduct rational hunting management, a certain number of wild animals must be harvested yearly. However, some countries have a problem with managing the harvested meat. An example is Poland, where game consumption is estimated at 0.08 kg/person/year. This situation leads to environmental pollution as a result of meat exports. The level of environmental pollution depends on the type of transport and distance. However, the use of meat in the country of harvesting would generate less pollution than its export. Three constructs were used in the study, which aimed to determine whether the respondents show food neophobia, whether they are willing to seek diversity in food, and what their attitudes towards game meat are. All the scales used were previously validated. Four-hundred and fifty-three questionnaires were collected using the PAPI method. It was found that the respondents showed ambivalent attitudes towards game meat to the greatest extent (76.6%), 16.34% had positive attitudes, and 7.06% had negative attitudes. It seems essential that most of the respondents were highly inclined to look for variety in food (55.85%). Regarding food neophobia, there were 51.43% of people with medium neophobia, while also many people with a low level of neophobia—43.05%. Such results allow speculation that the respondents are open to the new food, they are looking for it, and the low level of game meat consumption is primarily due to the lack of knowledge and awareness about the value of this meat.

## 1. Introduction

Hunting is highly controversial because most people have an emotional attitude towards hunting animals. The killing of animals for consumption, while an established way of providing food for humans, also poses ethical dilemmas [1]. It should be noted that in Poland, as well as in the world, there are three main groups of consumers who differ in their views on hunting, i.e., those with extremely negative, ambivalent, and highly positive attitudes. The diversity of consumer attitudes toward food is usually determined by many factors simultaneously, including their lifestyle, place of residence, diet, or generally understood worldview [2].

The main legal act regulating the hunting economy in Poland is the Hunting Law, adopted in 1995. Over the last quarter of a century, however, this law has evolved, new provisions were added to it, and the existing ones were reformulated. This document regulates many issues, although it is undoubtedly still not perfect, as indicated by the results of the research in [3]. The Hunting Law Act defines the hunting objectives in Poland, which include the following:

- protection, preservation of diversity, and management of wild game animal populations;
- protection and shaping of the natural environment to improve the living conditions of animals;
- achieving the highest possible individual condition and quality of trophies as well as the appropriate number of populations of individual game species while maintaining the balance of the natural environment;
- meeting social needs in the field of hunting, cultivating traditions, and promoting ethics and hunting culture [4].

The cited records show that hunting is primarily necessary to protect animals, keep them in good health, and cultivate centuries-old Polish traditions, and not only entertainment or ways to spend free time.

Apart from the arguments mentioned above, the results of the research by Schulp et al. [5] showed that statistically, in one year, each Pole eats about 0.08 kg of game meat represented by deer meat (*Cervus elaphus* L.) and roe deer (*Capreolus capreolus* L.), as well as wild boar (*Sus scrofa* L.) and hare (*Lepus europaeus* Pallas). For comparison, in France, this value is 5.7 kg of game/person/year, in Italy 3.8 kg, in Luxembourg 2.7 kg, in Sweden 2.63 kg, in Spain 1.36 kg, in the Czech Republic 1.1 kg, in Germany 0.9 kg, and in Slovenia 0.56 kg. In the light of these data, the question of the reasons for such a low level of game meat consumption in Poland becomes justified. Perhaps these are a lack of obtaining wild game, vegetarianism of Polish consumers, lack of consumer knowledge about wild animal meat, negative consumer attitudes towards this meat, food neophobia of Polish consumers, or perhaps a low tendency to look for variety in consumed food.

The considerations made in the article seem particularly important in the light of the concept of sustainable development, an essential element of which is sustainable consumption. Already in the 1930s, Aldo Leopold, the famous researcher and pioneer of the idea of hunting management, took up the topic of protection of land and green areas, which was very important for science and economy. He described it in detail in 1949 in the book entitled “A Sand County Almanac” in “Earth Ethics”. Aldo Leopold characterized protecting the land and green areas as a state of harmony between people and all the green spaces surrounding them. He also suggested the need to include soil, water, plants, and animals within the boundaries of the generally understood community, which would mean that all ethical issues should be related not only to humans (e.g., crime) but also to the environment (e.g., unjustified logging) [6].

In the following years, many authors popularized this or a similar approach. In 1987, the WHO published a report entitled “Our Common Future”, indicating, inter alia, the need to strive for sustainable development [7]. In turn, in 1992, the First Earth Summit in Rio de Janeiro was held, during which documents such as “Agenda 21” and the “Rio Declaration” were formulated. These documents defined the general assumptions of the idea of sustainable development, indicating that the use of existing resources should be such as to ensure their availability also for future generations. These records are not only up-to-date, but their importance is increasing in the light of the observed climate changes. These documents also include a chapter on sustainable consumption, which was initially defined as a change in consumption habits, including eating habits. It was then described as a new concept of wealth and prosperity, allowing humans to raise living standards by changing their lifestyles and also to be less dependent on the Earth’s limited resources. This way, potentially long-term goals have been set to change: consumer behavior, choices, expectations, and lifestyles [8].

Over the years, the definitions and assumptions of the idea of sustainable consumption have evolved. However, each of the currently available, regardless of the year of publication or source of its origin, is based on the original one, including the assumptions mentioned above. Sustainable consumption is the use of goods that meets basic needs and ensures a better quality of human life while minimizing the consumption of natural resources and the use of toxic materials, as well as reducing pollutant emissions and waste production throughout the product/service life cycle. These actions are aimed at reducing the risk to the needs of future generations [9].

The number of wild game animals in Poland is determined each year on 10 March. In 2022, data on big game were as follows: red deer 292.7 thousand heads, European roe deer 918.5 thousand heads, and wild boar 55.5 thousand heads. The population of the most important wild birds is estimated at 537 thousand heads of pheasants and 264.2 thousand heads of partridges. In turn, the amount of harvest determined after the entire hunting year (31 March–1 April) in the 2021/2022 hunting season was: 100.5 thousand red deer, 177.3 thousand heads of roe deer, 143.8 thousand heads of wild boars. The acquisition of wild birds was lower than that of big game. In the 2021/2022 hunting season, only 43.2 thousand pheasants and 1.4 thousand partridges were shot [10]. Assuming that the game was obtained correctly by a well-aimed shot in the chamber, in the case of roe deer, the slaughter efficiency is, on average, approx. 50%, red deer 47%, and wild boar 45%. If we simplify the estimates, we assume that each obtained deer weighed 15 kg, meaning 7.5 kg of meat was obtained in this way. This means that out of 177.3 thousand of roe deer, almost 1329 tonnes of meat was obtained. Similar estimates can be made for other animal species. Going further, assuming that the population of Poland is about 38 million people, and each consumes 0.08 kg, that is 3000 tonnes of meat per year. Since the roe deer meat itself is obtained in Poland at half of this value, the question of what happens to the remaining meat arises. Especially since red deer and wild boar undoubtedly weigh more than 15 kg. The answer to this question is quite simple: it is a valuable export commodity.

Exporting game meat from Poland to neighboring countries is a permanent phenomenon. In 2015, the total volume of game meat exports was 48 thousand tonnes. In 2020 it was 43.5 thousand tonnes, and in 2021 51 thousand tonnes. At the same time, it should be noted that Poland has drastically reduced the import of game meat in recent years. In 2015, 62 thousand tonnes of wild animal meat was imported to Poland. In 2018 it was 51 thousand tonnes. In 2020, however, it was only 20.5 thousand tonnes. This is undoubtedly a significant decrease, but it was probably caused by the COVID-19 pandemic, because in 2021, game exports increased to almost 30,000 tonnes [11].

In the light of the above-mentioned facts, another vital question can be formulated about the quality of Polish game meat, defined as a set of inherent properties of this meat. Polish game is a very desirable product on the European market. The meat of wild animals obtained in Poland is successfully exported, for example, to Belgium, where it costs about EUR 12 per kilogram. Another example of a high evaluation of the quality of Polish game meat is the activity of the Geti Wilba company, which has been supplying raw material (game) for the production of its preserves in Poland for many years. During the COVID-19 epidemic, when both hunting was cut and game export ceased, the company was on the verge of bankruptcy, despite its more than 50-year history. On the other hand, in Poland, one of the oldest, if not the oldest, companies dealing in the wild animal meat trade, Las-Skwierzyna-Gorzów, has suspended business operations [12]. On this occasion, it is also impossible not to mention the National Union of Game Exporters, operating since 2001, whose activity indicates the potential of game obtained in Poland. These examples show that the quality of Polish game meat is high, and the existing situation is undoubtedly a symptom of not using the potential of this valuable raw material.

In this context, another doubt arises as to whether Poles are a society with an eminently vegetarian model of nutrition. According to the data of the Central Statistical Office in 2020, this is not true, as the consumption of meat per capita in total households was 81.4 kg, comprising 43.4 kg pork, 2.2 kg beef, and 24.7 kg poultry meat, as well as 0.2 kg of other meat [10]. Based on these data, it can be concluded that a vegetarian diet is not a specific feature of Poles.

If Poles’ statistical consumption of meat, amounting to 81.4 kg of meat/person/year, were compared to the total volume of meat production, it could be concluded that it amounts to 3.072 million tonnes of meat. Such a large production of raw meat generates a huge environmental burden. At this point, another question arises: how does the current state relate to obtaining game in Poland and the world? By limiting the consumption of meat from farms (poultry, pork, beef) in favor of wild animal meat (by using it on the spot instead of exporting), it would be possible to achieve very beneficial effects for both meat consumers and the natural environment. Industrial livestock breeding is costly and time-consuming, but it also causes an excessive burden on the environment. In turn, the growth and development of wild animals are somehow included in the natural burden on the environment. Man does not (so far) influence the intensification of red deer or roe deer breeding. In some cases, it even limits it (wild boars). Therefore, the question arises as to why, by acquiring such a valuable raw material as game meat, Poland exports it instead of using it for the nutritional needs of Poles. It should be emphasized at this point that the export of any goods, and thus meat, also largely contributes to the burden on the environment and increases the carbon footprint. This is because it is transported frozen and therefore requires a constant energy supply to keep its temperature low and stable. According to the authors, the meat of wild animals is undoubtedly a valuable raw material, but also an ecological product desired by today’s consumers. Therefore, consuming it fresh would preserve its ecological nature. On the other hand, freezing, exporting, and further frozen storage generate costs, burden the environment (exhaust fumes, etc.), and therefore make the meat non-organic. Consequently, it would be worth considering how to use this raw material as soon as possible from the moment of obtaining it, i.e., by not exporting it or freezing it, but consuming it in the country of origin.

One of the most important psychological factors determining behaviors, including consumer’s eating behavior, are attitudes toward the object of consumption, treated as the resultant of three components: cognitive, emotional, and behavioral [13,14,15,16]. The cognitive component includes knowledge about the attitude object, the way this object is perceived, and its memory connotation. An attitude is an opinion about what a person thinks about reality without necessarily attracting or rejecting it. The emotional component, also referred to as the affective component, indicates an attractive or repulsive feeling that a person experiences toward the object of the attitude. This component is considered to be the heart of the attitude. In turn, the behavioral component of an attitude consists of intentions to behave, including the emotional attitude and knowledge about the subject of the attitude. Behavioral intentions indicate a propensity to behave towards the object of an attitude. Through the behavioral component, the attitude may induce an individual to approach the object or avoid it. Hence the theory that initially, there is a particular perception and interpretation of the object or situation constituting the object of the attitude (the cognitive component), then an emotional state (emotional component) appears, which stimulates one to act appropriately (the behavioral component) [17,18].

Considering the relationship of attitudes towards consumer behavior, it should be emphasized that the attitude identified in relation to a specific food does not have to be consistent with a particular behavior towards that food. Indeed, factors described as mediating variables or confounders are likely to effectively weaken the relationship between attitude and behavior. The disturbing factors include, first of all, economic limitations, situational factors, such as the influence of accompanying persons while shopping, as well as subjective norms and environmental factors, which include the impact of family members and the influence of the environment [19,20].

The above-mentioned considerations became the reason for undertaking research on the identification of respondents’ attitudes towards game meat as a factor constituting the intention to preserve the potential for the development of the meat market in Poland. However, the research results presented below are of a pilot nature; therefore, they will undoubtedly require further development.

In assumptions, this article aimed to review the potential and identify the real causes of the current state of game consumption in Poland. Simultaneously, we attempted to indicate the conditions for increasing the diversity of meat consumed by Poles. The specific purpose of the study was to identify the respondents’ attitudes towards game meat as a significant determinant of the consumption of wild animal meat, which was associated with the level of food neophobia and the tendency to seek food diversity. Therefore, it was assumed that these factors would constitute an important context in shaping attitudes towards game meat.

## 2. Materials and Methods

The study used a questionnaire consisting of 4 elements:

1. FNS scale (Food Neophobia Scale) [21];
2. VARSEEK scale (Variety Seeking Tendency Scale) [22];
3. An original questionnaire for identifying attitudes towards game meat using a 5-point Likert scale, which was named GMAS (Game Meat Attitude Scale) [23];
4. Sociodemographic questions.

The FNS and VARSEEK scales used in the study have been translated into Polish and edited and validated by Żyłka and Ocieczek [24]. The use of the FNS and VARSEEK scales in the study was not accidental. These scales complement each other perfectly.

The FNS scale allows determining whether the consumer’s attitudes are directed more towards the willingness to learn about new food products (neophilia) or staying with products known to the consumer (neophobia). This scale is significant because food is a product that a person cannot give up. Still, it is also a product that shapes certain attitudes, which is an important factor in determining certain behaviors towards it. That is why, from the point of view of a food producer operating in a competitive market, it is essential to know the rationale behind the consumer’s decisions related to the choice, purchase, and consumption of a specific food. Knowledge of these premises allows one to make rational decisions and therefore serves to manage the quality of the food product. When talking about quality management in the production and circulation of food, one should consider the implementation of the assumptions of quality management in obtaining raw materials, processing processes, and the logistics chain, as well as appropriate marketing strategies.

On the other hand, the VARSEEK scale, which in a way supplements the FNS scale, allows defining a consumer identified based on the FNS scale as having a neophilic attitude and as looking for diversity in food at a certain level [25]. Therefore, the use of these two scales, identifying the general level of consumer attitude towards food, in conjunction with the proprietary questionnaire for identifying attitudes towards game, seems to be necessary to achieve the goal set in the study.

An original questionnaire (GMAS) was used to identify the attitude towards game meat. The individual statements included in this questionnaire were used with a 5-point Likert scale. The use of a 5–point scale was justified by the results of studies which showed that the average perception capacity of the human mind might be limited. There are premises indicating limits to the amount of data the human mind can process [26]. The number of responses in the range of 7 ± 2 was considered the most justified in creating a cafeteria of discreetly differentiated responses. In the light of these results, for this study, the 5-point Likert scale was superior to other scales. Moreover, it was taken into account that in the Likert scale, categories are labeled by assigning names to individual categories, which makes it easier for the respondent to choose the best answer for their assessment. In this questionnaire, the most basic division of categories on the Likert scale was used: 1—“I completely disagree”, 2—“I disagree”, 3—“I neither agree nor disagree”, 4—“I agree”, 5—“I fully agree”. Using an excessively long scale in the present case could lead to a tendency to flatten it, combining several points on the scale into one [27].

Using three different constructs in one questionnaire may be risky. However, the authors took into account the common method bias of the constructs and tried to combine them in such a way as to avoid the most common problem, which is the respondent’s attempt to be consistent in the answers or filling in the questionnaire through the prism of its primary object, in this case, game. This risk did not arise with using the FNS and VARSEEK scales. In general, errors related to the test method constitute a significant problem as they are one of the primary sources of measurement errors. An error in selecting a method or conducting a study threatens the validity of inference in terms of relationships between the studied variables [28]. Hence, the justification for combining two popular and complementary constructs often used jointly at the beginning and only at the end of the proprietary questionnaire focused on identifying consumers’ attitudes towards game.

It is also necessary to explain why the authors believe that game meat should be equated not only with the concept of traditional food but also with the concept of novel food. Mesinger and Ocieczek [29] stated that traditional food is not only consumed by specific communities in specific cultural conditions. Products consumed by previous generations but gradually replaced by innovations appearing over time, which return to the market in their original form after a relatively long time as a novelty, can also be considered a traditional food. Such a situation can now be talked about in Poland concerning game meat, which is referred to as the second life cycle of this product. Since its introduction, such a product is still present on the market, but in different amounts, and the consumers’ interest in this product is highly diversified. Hence the conclusion is that wild animal meat is currently experiencing its second life cycle, and this is the perfect moment for changes in both the hunting management system and the game meat. Unfamiliarity with game by consumers makes it a new product and justifies using the FNS and VARSEEK scales for a comprehensive study of attitudes towards it.

### 2.1. Food Neophobia Scale

Table 1 shows the statements that make up the FNS scale used in the survey. Marking (N) on the statement indicates that it was re-coded in the calculation procedure because its sentiment was negative [21].

### 2.2. Variety Seeking Tendency Scale

Table 2 lists the statements used in the VARSEEK scale in this questionnaire. Marking (N) next to the statement indicates the necessity to re-code it due to the negative tone [22].

### 2.3. Game Meat Attitude Scale

The original questionnaire on the identification of attitudes included statements relating to the cognitive (P), emotional (E), and behavioral (B) components. The statements and the assignment to a specific component are summarized in Table 3. In addition, statements where it was necessary to recode due to the negative overtones were marked (N) [23].

The GMAS construct presented below was developed by Mesinger et al. [23]. Based on their knowledge and literature data, the authors of the construct identified various potential causes that could condition attitudes toward game meat. It was found that the factors that consumers may consider important are price, availability, sensory characteristics of meat, difficulty in culinary processing, product safety, and nutritional value. 

The GMAS scale used for the purpose of this study was previously validated in terms of its possible use. Content validation, validation of the response process (the so-called face validation), statistical validation (Principal Factor Analysis (PCA) using Varimax orthogonal rotation), and reliability testing using Cronbach’s alpha were carried out for this scale [23].

### 2.4. Statistical Analysis of the Obtained Results

The first essential element of the statistical analysis of the obtained results was the determination of the reliability coefficient—Cronbach’s Alpha. The higher the test’s internal consistency, the higher the Cronbach’s Alpha value. This statistic assumes various minimal values; usually, the required value is above 0.60 [30].

The chi-square (χ2) test of independence was used to analyze the obtained results. It is a test used to assess the response frequency distribution conditioned by a factor differentiating the studied population. The independence test allows stating whether the analyzed distribution of results is conditioned by a specific differentiating factor [31]. The factors differentiating the respondents were the following sociodemographic data: gender, age, education, financial situation, subjective assessment of the diet, and eating game meat (at any time in life).

### 2.5. Characteristics of the Researched Population

The questionnaires were handed over to random respondents in the number of 482. After eliminating incomplete or incorrectly completed questionnaires, 453 were used for the analysis. The study group did not represent the entire population of young Polish consumers. The essence of the study was only to test the proprietary part of the questionnaire and to obtain data for the initial characterization of respondents as potential game consumers. It should be emphasized, however, that implementing a similar study, also based on the results obtained from a random sample, will not constitute a basis for forecasting trends and, therefore, for forecasting future consumer behavior. The law of large numbers states that the occurrence (or non-occurrence) of an event in the future is as probable as it was probable that it would occur (or not) in the past under similar conditions. In the case of social phenomena, this condition is unrealistic. Therefore, the study presented in this study was of a pilot nature. It will require re-conducting in a much larger territorial area and a larger sample to ensure greater reliability of the obtained characteristics and not to obtain a basis for forecasting trends [32].

The study group consisted of relatively young respondents, aged 18–40, with different levels of education. The choice of such a population as a research sample was not accidental. It was conditioned by the fact that, according to the opinions of various groups of researchers, it is a group of consumers characterized by low consumption of game meat [33]. At the same time, it is a group susceptible to changes in eating behavior, looking for new products, and sensitive to changes in the natural environment and climate [34]. Moreover, it has not been sufficiently researched in terms of attitudes towards game meat and their relationship to the consumption of this raw material. Detailed data, taking into account the percentage distribution of the respondents’ differentiation, are presented in Table 4.

The survey was conducted using the PAPI (Paper and Pen Personal Interview) method, where a properly trained interviewer interviewed each respondent personally. This method allows for proper control of the test sample and achieves high accuracy of the obtained results. The disadvantage of this method is the long duration of the research and the respondent’s belief that they are not anonymous. However, the conducted study was fully anonymous, in line with the stipulations of the Helsinki Declaration [35].

People taking part in the study were randomly selected from people living in the Tri-City (northern Poland). A stratified random selection of the test sample was used. The study area was divided into three layers: the first was Gdańsk, the second was Gdynia, and the third was Sopot. In each of the layers, two Biedronka stores located in the city center and on its outskirts were selected, where the study was conducted. As a result, the research involved young people living in the Tri-City, who actively shop for groceries, thus demonstrating specific consumer behaviors and attitudes towards selected or rejected goods. The main study sample was not representative of the general population of young people living in Poland.

The survey was conducted from October to December 2019 in the Pomeranian Voivodeship (cross-sectional study).

## 3. Results

The results of each construct were analyzed separately. The reliability analysis of the obtained results for each construct consisted of recoding negative statements. Statements 1, 4, 6, and 10 were re-coded on the FNS scale, statement 7 on the VARSEEK scale, and statements 2, 5, 8, and 10 in the case of the original GMAS questionnaire. Moreover, the scale’s reliability was determined by calculating Cronbach’s alpha coefficient.

The set of statements proposed in the proprietary questionnaire (GMAS) and the described analytical procedure indicated that the more points the respondent scored, the more positive their attitude towards the meat of wild animals was. In the case of this questionnaire, the score could be from 10 to 50 points. Therefore, the average results obtained for this construct of 34.09 indicated a tendency towards positive attitudes towards game.

The preliminary analysis of the results showed that the studied group of respondents was characterized by an average tendency to look for a variety of food and an ambivalent attitude towards new/unknown food, which means that the level of food neophobia in this group was not high. The results of the reliability analysis for individual constructs are summarized in Table 5.

For each of the constructs, the re-coding of points was used in the case of negative statements, which made it possible to estimate the mean values obtained by individual respondents. In this way, the respondents were divided into three groups. Due to the fact that in the VARSEEK construct, there is a smaller number of items (8) than in the GMAS and FNS (10) constructs, it was decided not to add up the scores obtained by the respondents. Instead, the average of the points obtained for each scale was calculated for each respondent. This allowed for the adoption of one variant of grouping the respondents, which made the results obtained in individual scales possible to correlate with each other, looking for potential relationships between FNS, VARSEEK, and GMAS.

For each construct, each respondent could score from 1.0 to 5.0 points (based on the mean calculation). Therefore, the division into groups as closely related as possible was used. Namely, in group 1, there were people who scored 1.0–2.2 points, in group 2–2.3–3.7 points, and in group 3–3.8–5.0 points. This means that the first group consisted of people with negative attitudes towards game meat in the case of the GMAS construct, in the case of the FNS construct—people with neophilic attitudes, and in the case of the VARSEEK construct—people with a low tendency to seek diversity in food. In the case of all constructs, the second group consisted of people with ambivalent attitudes. In turn, the third group in the case of FNS comprised people with food neophobia, in the GMAS construct—people with positive attitudes towards game, and in the VARSEEK construct—people willing to look for diversity in food. The number of people in each group and the results obtained are presented in the following parts of this paper.

### 3.1. FNS

**Figure 1:**
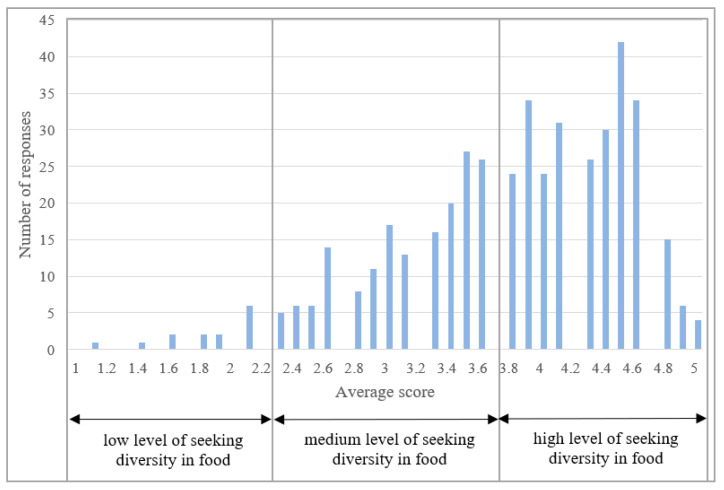
Distribution of neophobia levels for the FNS scale within the study group.

Next, the response distribution was analyzed concerning the FNS construct. The reliability of the FNS scale was estimated at 0.8443. It is a value that indicates the correct reliability of the scale. Figure 1 shows the distribution of points obtained by respondents in the FNS construct. On the other hand, Table 6 presents the quantitative and percentage breakdown of the respondents depending on the group.

The analysis of the results and calculations indicate the existence of a very large group of respondents who show ambivalent attitudes, which in the case of the FNS scale means that an average level of neophobia characterizes them. At the same time, the obtained results indicated that only 5.52% of the studied population was classified in the third group, which showed a high level of food neophobia. Therefore, the respondents are characterized by a more neophobic than neophobic attitude. Table 7 presents the analysis of χ2 respondents’ answers in the FNS construct regarding individual statements. Moreover, an analysis of the diversity of responses in terms of various analyzed sociodemographic features is also presented.

Based on the data collected in Table 7, it was found that gender was a factor that statistically significantly differentiated the respondents’ responses to statements 4 and 9. Women had a more positive perception of food from different countries and showed a more positive attitude to the statement, “I will eat almost everything”. On the other hand, age and education were not factors differentiating the respondents’ attitudes towards any statement. The financial situation significantly differentiated the respondents’ answers to statement 6. However, people who indicated a good financial situation rated the issue of new products consumed during special events the highest. Subjective evaluation of the diet differentiated the subjects in terms of statements 1, 4, 5, 6, 7, and 10. Noteworthy are the statistically significant differences in the respondents’ responses on the FNS scale depending on whether they had ever eaten game. In this classification, statistically significant differences were found in the case of items 1, 2, 3, 4, 6, 8, 9, and 10. It should be noted that a higher level of neophobia characterized the respondents who never consumed game meat. The factor that did not differentiate the respondents in a statistically significant way in terms of any item of the FNS scale was age.

Table 8 presents the analysis of the obtained results in terms of the level of neophobia in the case of differentiation of groups based on sociodemographic features and the statistical significance of these differences.

Among men, a lower percentage of people with medium and high levels of neophobia was found and higher in the case of low levels than among women. The analysis of the number of points obtained by the respondents in the context of their education allowed for the conclusion that people with higher education have more neophilic attitudes than people with secondary education. However, the biggest difference was found when comparing respondents’ responses based on whether they had ever eaten game in their lifetime. In this case, people who had ever eaten game were definitely more open to new and unknown foods. In contrast, people who had never eaten game had somewhat ambivalent or negative attitudes in the context of the FNS construct. It should be noted that this was the only feature differentiating the entire analyzed population in terms of the FNS scale, which showed statistically significant differences.

The analysis of the χ2 test results in terms of gender, age, education and game consumption, taking into account the previously assumed ranges of the average number of points as a differentiating factor, allowed for the conclusion that gender, age, and education did not differentiate the respondents in terms of their level of neophobia. On the other hand, statistically significantly differentiating between respondents was whether they had ever eaten game.

### 3.2. VARSEEK

**Figure 2:**
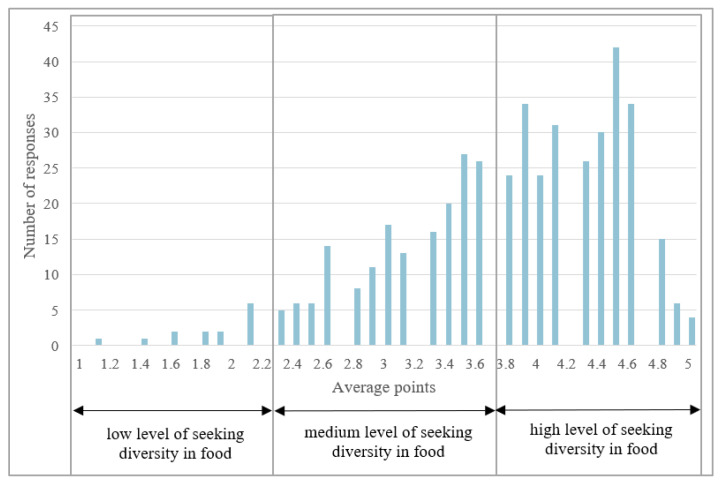
Distribution of the levels of the tendency to look for diversity in food within the studied group.

The distribution of responses obtained in the VARSEEK construct also strengthened the preliminary conclusions drawn from the analysis of the results from the FNS construct. Figure 2 shows the distribution of points obtained by the respondents in the VARSEEK construct. On the other hand, Table 9 presents the general characteristics of the studied population in terms of assigning respondents to three groups.

The left-skewness found based on the obtained distribution of mean points, which the respondents received, proves the existence of a high level of their tendency to search for a variety of food. This justifies the preliminary conclusion that consumers are open to unfamiliar products. Undoubtedly, wild animal meat can be considered such a product because many people rarely meet it or only on the occasion of special meetings. At the same time, people do not eat it at home. The identified consumer openness towards novel foods may mean that the appropriate method of managing the production of processed game meat and managing the quality of such products with the simultaneous use of marketing tools and educational campaigns may directly match the expectations and tastes of consumers. This, in turn, may have the desired effect of accepting this meat as part of the daily diet of many consumers. Table 10 presents the results of the analysis of the mean values of points for individual statements in the VARSEEK scale, the statistical significance of differences and differences within particular sociodemographic features.

In the case of the analysis of the scale of the tendency to look for diversity in food, gender differentiated the respondents only in the case of statement 7. Namely, women were more eager to indicate that they prefer to eat the products to which they are used to. Age was not a statistically significant differentiating factor in the case of any of the findings. Education differentiated the respondents in a statistically significant way in terms of items 3 and 4. People with secondary education were more optimistic about the unknown dishes and those consumed in other countries. The financial situation allowed indicating the diversity of the respondents in the case of statements 2, 6, and 7. People describing their financial situation as good most often indicated that they are willing to try to prepare food according to new recipes. People who indicated that their financial situation was bad most often suggested that they were interested in unfamiliar food products. On the other hand, people whose financial situation is sufficient most often stated that they prefer to eat the products to which they are used to. The subjective assessment of the diet allowed finding statistically significant differences in the respondents’ assessments for all the VARSEEK scale statements. People describing their diet as very good were characterized by the highest tendency to look for variety in food. This undoubtedly points to the fact that nutritionally conscious people, familiar with nutrition principles, realize that the diet should be as varied as possible.

Whether the respondents had ever eaten game meat revealed statistically significant differences in responses for most of the statements (statements 1, 3, 4, 5, 6, 7, and 8) in terms of the level of the tendency to look for diversity in food.

Table 11 compares the statistical significance of the low, medium, and high propensity to seek a variety of food respondents regarding gender, age, education, and whether they had ever eaten game.

Based on the obtained results (Table 11), it was found that only the consumption of game was a statistically significant differentiating feature in the study population in terms of the tendency to seek a variety of food. Subjects who had never eaten game were significantly less likely to seek variety in their food than those who ate wild animal meat at least once in their lives. Additionally, a significantly smaller percentage of respondents with higher education showed a lack of inclination to look for a variety of food compared with people with secondary education. Perhaps this means that the level of education affects the ability to take a particular position. People aged 21–40 significantly more often showed a high inclination to seek variety in food than people under 20 years of age. On the other hand, this younger group of respondents was characterized by ambivalence rather than a low propensity to search for various foods. Men exhibited extreme, negative, or positive attitudes, while in the case of women, a higher percentage of people with ambivalent attitudes was recorded.

### 3.3. GMAS

**Figure 3:**
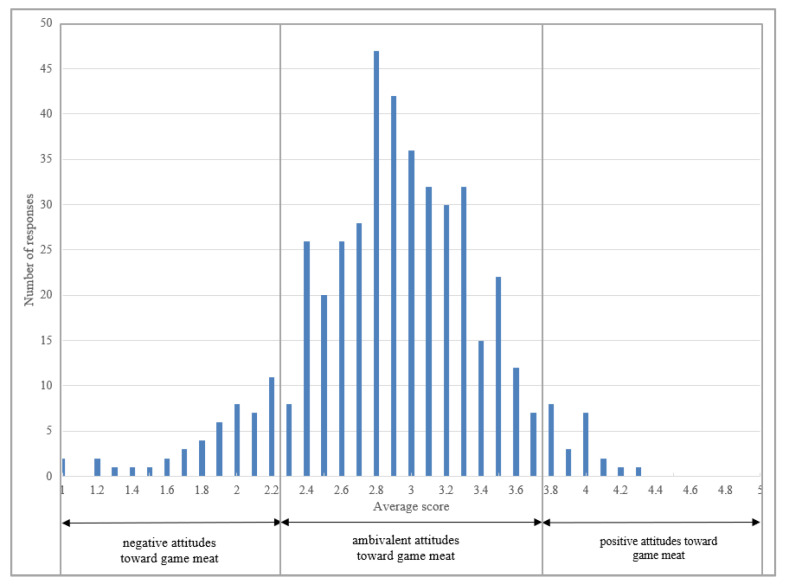
Distribution of attitudes towards game meat within the study group.

The descriptive statistics presented in Figure 3 show the distribution of points obtained by the respondents in the construction of the proprietary questionnaire on attitudes towards game meat. On the other hand, Table 12 presents the general characteristics of the respondents in the scope of previously determined groups with a defined average number of points.

The analysis of the data presented in Figure 3 shows that the dominant part of the respondents is characterized by ambivalent attitudes towards game, which was reflected in the average value estimated based on its individual components. At the same time, it should be stated that the existence of noticeable differences related to individual components was identified, e.g., in the case of the behavioral component, the respondents showed extremely positive attitudes. In contrast, concerning the cognitive component they showed extremely negative attitudes. It can be stated that the surveyed group of respondents was characterized by a low level of knowledge about game meat safety and its nutritional value. Consequently, the obtained resultant values indicated the existence of ambivalent attitudes as dominant and specific for the examined group of respondents. The obtained result requires further, in-depth analysis and suggests the need for another study in a broader population.

Table 13 presents the results of the analysis of the mean values of points for individual statements in the GMAS scale, the statistical significance of the differences, and the differences related to the particular sociodemographic features.

The descriptive statistics presented in Table 13 determined the GMAS construct, suggesting that gender differentiated respondents in a statistically significant way for statements 1, 3, and 4. Therefore, it can be concluded that men are more aware of the health benefits of game and game consumption. They would try game meat more willingly than women if its availability and price were more favorable. It should be emphasized, however, that the average number of points obtained by men in terms of these statements did not exceed the value of 3.59. Therefore, it can be assumed that further education of the respondents of both genders in this respect is necessary because their current level of knowledge seems insufficient.

The analysis of the results allowed for the conclusion that age and the financial situation did not differentiate the respondents in terms of any statement of the GMAS construct. Education allowed determining statistically significant differences for items 6 and 8. People with higher education showed awareness of the existence of differences between the properties of meat from wild and farm animals. People with secondary education more often described wild animal meat as insufficiently tested.

The analysis of the responses in the context of subjective diet assessment showed that the responses of the respondents were statistically significantly differentiated in the case of statements 1, 3, 4, and 6. The respondents describing their diet as very good were characterized by the greatest knowledge about the health benefits of the consumption of game and its difference compared to the meat of farm animals. On the other hand, those who declared their diet was good most positively approached the idea of eating game if it would be cheaper and more readily available in retail stores.

A history of consumption of wild animal meat at any time in life showed statistically significant differences for items 1, 2, 3, 4, 5, 7, 8, and 9. People who consumed game had a higher score for all of the above-mentioned statements. Therefore, it can be concluded that if someone who ate game meat were more aware of its health benefits, they would be more open to consuming it more often in the case of a lower price and better availability on the market. In addition, respondents who had ever eaten game in their lifetime are more aware that game is different from the meat of farm animals and that it is lean and contains a lot of minerals. In turn, people who had never eaten this meat in their lives showed a lower level of knowledge regarding these factors.

Summing up, it can be stated that respondents’ attitudes towards game depend largely on its consumption. However, the level of knowledge and awareness of the benefits of eating this meat still seems insufficient, regardless of the feature differentiating the surveyed group of respondents in which their responses were analyzed.

On the other hand, the analysis of the results in the manner presented in Table 14, taking into account the division of respondents into groups with negative, ambivalent, and positive attitudes towards game in terms of gender, age, and education, allows for the identification of statistically significant differences determined by sex and game consumption.

Women much more often showed negative attitudes towards game, while men more often showed ambivalent or positive attitudes. This indicates that men are more open to meat, such as game. Age and education did not differentiate the respondents’ attitudes in a statistically significant manner. However, eating wild animal meat differentiated the respondents’ attitudes in a highly statistically significant way. In total, 13.79% of respondents who had never eaten the meat of wild animals showed negative attitudes towards it, while those who had contact was only 3.9%. This information is crucial because it indicates the lack of openness of people who have not eaten game or their lack of knowledge. Regardless of the reasons for such a state of affairs, it is undoubtedly necessary to educate consumers constantly.

## 4. Discussion

The discussion of the results obtained in this study is based on comparing them with the results of English-language studies describing the conditions of game consumption by consumers from different parts of the world. The possibility of conducting a discussion based on the results of Polish respondents is minimal. The works of Polish researchers are published mainly in Polish and were most often carried out on small groups of respondents. There is also little work on the attitudes and behavior of Polish consumers towards game published in English, but it is from them that the discussion of the results should begin.

Kwiecińska et al. [36] attempted to identify factors influencing consumer acceptance or rejection of game. The above-mentioned studies analyzed the responses of 1000 respondents, and the basic conclusion was, as in this study, that men are more open and willing to change the meat they eat. Moreover, it was found that consumers would be more likely to eat game if its quality and availability were better. This means that consumers show a certain degree of openness to game meat but are also concerned about its sensory characteristics. Moreover, these authors stated that game consumption in highly developed countries is as high as 6 kg/person/year, which suggests that perhaps Poland is not yet at the right stage of economic and social development to accept this type of meat.

In 2020, Niewiadomska et al. [37] conducted research to identify factors that encourage consumers to include game in their diet. The inference was made based on the responses of 450 respondents, and the Food Choice Questionnaire Item used in the study was divided into the part related to emotional and rational motives that encourage the consumption of game meat. Considering the results of numerous studies showing that attitudes are a factor predisposing to specific behaviors, understanding them is extremely important in searching for possible changes in consumers’ eating behavior in the future. The conclusions from the cited studies indicated the following:

1. The emotional motives of consumers to reach for game are weight control and knowledge of the product,
2. Rational motives for consumers to reach for game meat are taste, ease of preparation, product origin (domestic product), nutritional value, and a small amount of fat [37].

The last available publication on the discussed issue in Poland was the analysis carried out by Krokowska-Paluszak et al. [38]. However, the research did not directly cover attitudes towards meat. The preliminary assumption of this work was that people who know at least one hunter are more likely to show positive attitudes towards hunting and the willingness to eat game. In total, 486 respondents took part in the research. The basic conclusion was that consumers had a deficient knowledge and awareness about the necessity of hunting. The respondents perceived hunting only in the context of recreation and sport. This conclusion is analogous to the conclusion of this study, which allowed for the conclusion that it is necessary to conduct reliable and emotionally free educational activities concerning the nutritional and health values of wild animal meat. Undoubtedly, such activity is part of another important area of modern human life, namely health management. Moreover, educational activities should be based not only on showing the advantages of wild animal meat as food but also on the necessity of hunting to maintain the biological balance in forest ecosystems truncated by humans. Research by Krokowska-Paluszak et al. [38] also showed that the price and availability of wild animal meat is a problem for consumers. These results correlate with what was shown based on the analysis of the results presented in this paper. However, this issue requires more detailed research. Krokowska-Paluszak et al. [38] also found that acquaintance with a hunter, or having a hunter in the family or among friends, has a very positive effect on attitudes towards hunting in general.

Research conducted in South Africa has indicated the need to determine how the consumer makes decisions about eating game meat before implementing any corrective measures to increase its consumption. Radder [39] proposed a model of such a procedure, in which she distinguished five main stages: 1. awareness and recognition of the need; 2. interest, and information on harvesting and processing; 3. trial use of the product and its evaluation; 4. confirmation and initial approval of the product; 5. continued product rollout. The stages identified by her also indicate essential activities that should be considered when preparing any educational model or conducting research related to the dissemination of game among consumers. In the light of the results of the research by Radder [39] and taking into account their relationship with the results of this study, it is possible to formulate preliminary conclusions and suggestions regarding the necessary changes in the management of the game product and plan consumer education activities.

The research, being the essence of this study, included the identification of respondents’ attitudes towards such statements as (2)—The meat of wild animals is heavily contaminated with heavy metals; (5)—Eating wild boar meat is equivalent to infection with trichinella; and (8)—Game is a meat that has not been sufficiently researched concerning the safety of game consumption. The respondents assessed that the meat of wild animals is highly contaminated with heavy metals at 3.58/5.0. On the other hand, the results of the research by Mesinger and Ocieczek [40] indicated that in the case of red deer meat obtained in the northern region of Poland, the level of heavy metals, i.e., arsenic, cadmium, lead, and mercury, was below the limit of quantification. Wild animal meat should, therefore, not be viewed as dangerous due to the level of contamination with heavy metals. The respondents also often indicated that eating wild boar meat is tantamount to infection with trichinella (4.02/5.0). Meanwhile, meat, including wild animals, intended for human consumption is subjected to numerous laboratory tests and is subject to veterinary assessment. In Poland, game meat is no longer subject to a separate act when it comes to placing it on the market. It is treated like any other meat, so it is subject to the exact requirements for meat available in supermarkets. It results from the Act of 16 December 2005, on animal products [41]. Despite the regulations in force, the respondents were not convinced about game meat safety because the statement that it is insufficiently tested meat obtained a result of 3.39/5.0. This, in turn, allows concluding that there is a need to educate consumers about the safety of wild animal meat because the respondents did not demonstrate knowledge in this area.

The study on the assessment of attitudes towards safety related to game consumption was carried out by Owens and Branham [42] on a group of 79 respondents from San Angelo. Most of the respondents dealt with hunting and assessed their knowledge of game safety at a very high level, which was very surprising in the context of mistakes made in the process of gaining game, of which they were unaware of. These, in turn, were important for the safety and quality of meat, such as storing meat at too high a temperature or cleaning carcasses with paper towels.

The safety of meat from wild and farmed animals released to the market must be adequately tested. Therefore, the meat itself, if obtained from a reliable source, should not threaten the consumer’s health [40]. Nevertheless, wild animals can be a factor in the spread of zoonoses. Consequently, sometimes it is necessary to dispose of part of the carcasses, which is undoubtedly not ecological. The literature emphasizes that it is unfavorable to conduct extensive grazing of farm animals in the vicinity of natural habitats of wild animals. Studies carried out in south-central Spain have shown that the use of the same or very close habitats alternately in short intervals by wild animals (birds, wild boars, deer) and farm animals (cattle, pigs) can be the cause of the spread of tuberculosis. For this reason, it is undoubtedly necessary to properly protect grazing places for farm animals against access by wild animals, which will reduce the risk associated with the transmission of microorganisms [43].

Changes in agricultural practices, changes in the management of wild animals, their fattening, and also the creation of new human habitats in place of forests can potentially lead to the emergence of pathogens capable of infecting several species. Therefore, it is crucial to conduct long-term, multi-year studies on changes in the health of wild and farmed animals. Only such a comprehensive approach will allow for determining the factors affecting the transmission of diseases between species, and also what consequences it has for human health. It is necessary to regularly examine the animals, assess their health, and examine the quality and health of the meat obtained. This is due to the fact that there are already pathogens that can be transmitted between wild and farmed animals, e.g., African Swine Fever Virus can be transferred between wild boar and pigs if the animals are exposed to contact or function in the same or close area [44]. Therefore, publicizing that game safety tests are regularly conducted, and scientific research on the transmission of pathogens could also improve consumer attitudes in this regard.

The literature widely discusses the subject of low fat content in wild game meat, as well as the presence of bioactive ingredients in this product [37,45,46,47]. In turn, Soriano and Sanchez-Garcia [48] conducted an analysis of the nutritional value of wild animal meat obtained in Central and Mediterranean Europe. Their research covered a wide range of species, but also the variability of the climate and the type of vegetating plants. It was found that apart from the factors mentioned above, age, sex, health condition, and the hunting period greatly influenced the game nutritional value. Based on the obtained results, it was found that the most popular types of wild game meats (red deer, roe deer, fallow deer, wild boar, and hare) contained very little fat (3–4%), a lot of complete protein (20–26%), and good nutritionally fatty acid profiles. Therefore, values describing the content of essential ingredients in game meat are much more desirable than those that can be identified in meat from livestock. In addition, wild ruminant meat contains a lot of potassium, phosphorus, zinc, and iron, as well as vitamins E and B. Meanwhile, respondents participating in this study were not convinced whether wild animal meat contains more or fewer minerals than meat from farm animals. Therefore, consumer education in the field of game values is also a necessity.

## 5. Conclusions

Poland is one of the leading producers and exporters of game meat in Europe. However, the export of game meat in the light of the idea of sustainable development and sustainable consumption appears to be essentially non-ecological and irrational. Game, as a raw material that meets almost all the conditions, apart from official certification, of organic raw material, the production of which is not influenced by humans, through its export, contributes to the burden on the natural environment. Thus, it loses the value of pure environmental friendliness. Game is essentially a by-product of the hunting economy rather than its purpose. The development of wild animals, whose meat is intended for human consumption, is appropriately included in the functioning of the natural environment. Therefore, it does not result in any additional abnormal environmental burden. However, its exports already contribute to such a burden. In this light, the question arises: why is the export of game meat obtained in Poland so large, and what are the conditions for this activity?

Low consumer awareness of the value of this meat and low propensity to buy it forces its export to countries where the tradition of game consumption has been preserved, despite the high price. Thus, the question arises of whether it would not be wiser to apply educational campaigns in the country of obtaining meat and trying to use them on the domestic market.

The characteristics of the respondents obtained in the research with the use of constructs indicate that they are characterized by a sufficiently high level of neophilia and are inclined to seek diversity in food. At the same time, they are ambivalent towards game. Therefore, it seems legitimate to say that adequately implemented education will allow for forming positive attitudes based on the identified ambivalent attitudes. This, in turn, can be seen as an essential factor leading to a change in behavior towards an increased interest in purchasing and consuming game as an alternative to meat from slaughtered animals. This change may be an essential element in rationalizing the process of managing this product and hunting and, consequently, the implementation of sustainable consumption and sustainable development.

## Figures and Tables

**Table 1:** List of statements included in the FNS scale.

Statement No.	Statement
1.	I am constantly sampling new and different foods.
2.	I don’t trust new foods (N)
3.	If I don’t know what a food is, I won’t try it. (N)
4.	I like foods from different cultures.
5.	Ethnic food looks too weird to eat. (N)
6.	At dinner parties, I will try new foods.
7.	I am afraid to eat things I have never had before. (N)
8.	I am very particular about the foods I eat. (N)
9.	I will eat almost anything.
10.	I like to try new ethnic restaurants.

Possible answers: 1—“I completely disagree”, 2—“I disagree”, 3—“I neither agree nor disagree”, 4—“I agree”, 5—“I completely agree”; (N)—Recoded statement.

**Table 2:** List of statements included in the VARSEEK scale.

Statement No.	Statement
1.	When I eat out, I like to try the most unusual items, even if I am not sure I would like them.
2.	While preparing foods or snacks, I like to try out new recipes.
3.	I think it is fun to try out food items one is not familiar with.
4.	I am eager to know what kind of foods people from other countries eat.
5.	I like to eat exotic foods.
6.	Items on the menu that I am unfamiliar with make me curious.
7.	I prefer to eat food products I am used to. (N)
8.	I am curious about food products I am not familiar with.

Possible answers: 1—“I completely disagree”, 2—“I disagree”, 3—“I neither agree nor disagree”, 4—“I agree”, 5—“I completely agree”; (N)—Re-coded statement.

**Table 3:** List of statements for the identification of respondents’ attitudes towards game (GMAS).

No.	Statement	Component
1.	There are health benefits to consuming wild animal meat.	P
2.	Wild animal meat is heavily contaminated with heavy metals. (N)	P
3.	I’d like to try game meat if it was cheaper.	B
4.	If it were widely available in stores, I’d like to try game.	B
5.	Eating wild boar meat is synonymous with trichinella infection. (N)	B
6.	The nutritional value of wild animal meat is the same as that of farm animals.	P
7.	Game meat is lean.	P
8.	Game is meat that has not been sufficiently researched. (N)	E
9.	Game meat contains more minerals than livestock meat.	P
10.	Game meat is difficult to prepare. (N)	P

Possible answers: 1—“I completely disagree”, 2—“I disagree”, 3—“I neither agree nor disagree”, 4—“I agree”, 5—“I completely agree”; (N)—Recoded statement.

**Table 4:** Distribution of sociodemographic features in the study group, taking into account the % of all respondents.

Indicator	Characteristic	No. of Respondents	% of the Group (*n* = 453)
Sex	Women	316	69.76%
	Men	137	30.24%
Age	<20	49	10.82%
	21–40	404	89.18%
Education	High school	240	52.99%
	University	213	47.01%
Financial situation	Bad	6	1.32%
	Sufficient	114	25.17%
	Good	272	60.04%
	Very good	61	13.47%
Subjective evaluation of nutrition	Very bad	5	1.10%
	Bad	19	4.19%
	Common	192	42.38%
	Good	195	43.05%
	Very good	42	9.28%
Have you ever eaten game?	Yes	308	68%
	No	145	32%

**Table 5:** Results of the reliability analysis of the constructs used in the study.

Construct	Mean	SD	N Valid	Alfa Cronbach	Standardized Alpha	Average Correlation between Items
FNS	2.45	0.77430	453	0.8443	0.8493	0.3677
VARSEEK	2.96	0.6999	453	0.9033	0.9050	0.5555
GMAS (Game Meat Attitude Scale)	3.41	0.6439	453	0.6968	0.6857	0.1813

**Table 6:** Distribution of the population for the FNS scale.

	Average Score	Group Size	% of the Population
Group I (low level of neophobia)	1.0–2.2	195	43.05%
Group II (average level of neophobia)	2.3–3.7	233	51.43%
Group III (high level of neophobia)	3.8–5.0	25	5.52%

**Table 7:** Compatibility of the respondents’ opinions with the statements of the FNS scale (average values).

Statement	1.	2.	3.	4.	5.	6.	7.	8.	9.	10.
Overall average for the statement	2.88	2.63	2.28	2.18	1.97	1.98	2.30	2.96	3.01	2.31
Sex										
*p-*value				*p* = 0.0150					*p* = 0.0008	
Women	2.88	2.59	2.24	2.25	1.95	1.97	2.31	3.05	3.19	2.37
Men	2.90	2.72	2.35	2.01	2.01	2.01	2.30	2.77	2.61	2.18
Age										
*p-*value										
≤20	2.73	2.49	2.37	1.88	1.78	1.98	2.24	3.33	3.18	2.24
21–40	2.90	2.64	2.26	2.22	1.99	1.98	2.31	2.92	2.99	2.32
Education										
*p-*value										
High school	2.95	2.66	2.32	2.15	2.04	2.05	2.31	3.02	2.97	2.33
University	2.81	2.59	2.23	2.22	1.89	1.90	2.30	2.91	3.06	2.30
Financial situation										
*p-*value						*p* = 0.0170				
Very good	2.62	2.62	2.16	1.87	1.97	1.57	2.25	3.28	3.03	2.00
Good	2.93	2.64	2.33	2.22	1.97	2.08	2.35	2.96	3.00	2.34
Sufficient	2.90	2.62	2.23	2.28	2.00	1.96	2.26	2.83	3.02	2.39
Bad	3.17	2.00	2.00	1.67	1.33	1.83	1.83	2.67	3.00	2.67
Subjective evaluation of the way of nutrition										
*p-*value	*p* = 0.0000			*p* = 0.0000	*p* = 0.0420	*p* = 0.0000	*p* = 0.0000			*p* = 0.0000
Very good	2.14	2.12	1.83	1.64	1.67	1.50	1.67	3.10	2.74	1.69
Good	2.79	2.58	2.27	2.02	1.87	1.91	2.17	2.89	3.03	2.18
Common	3.09	2.80	2.35	2.43	2.11	2.16	2.51	2.99	3.09	2.53
Bad	3.29	2.50	2.50	2.46	2.17	1.96	2.92	3.13	2.75	2.67
Game consumption										
*p-*value	*p* = 0.0120	*p* = 0.0180	*p* = 0.0059	*p* = 0.0047		*p* = 0.0000		*p* = 0.0020	*p* = 0.0000	*p* = 0.0023
Yes	2.77	2.53	2.16	2.06	1.96	1.82	2.27	2.79	2.75	2.18
No	3.12	2.83	2.53	2.45	1.99	2.32	2.37	3.34	3.56	2.59

*p-*value was noted in the table only if there were statistically significant differences between groups.

**Table 8:** Characteristics of the level of neophobia in particular groups of respondents.

The Level of Neophobia	Sex		Age		Education		Game Consumption		Overall
	**Statistical Differences (*p*-value)**								
							***p* = 0.0477**		
	Women	Men	≤20	21–40	High School	University	Yes	No	
Low	41.77%	45.99%	42.86%	43.07%	42.08%	44.13%	48.05%	32.41%	43.05%
Medium	52.22%	49.64%	55.10%	50.99%	51.67%	51.17%	48.05%	58.62%	51.43%
High	6.01%	4.38%	2.04%	5.94%	6.25%	4.69%	3.90%	8.97%	5.52%
Overall	100%	100%	100%	100%	100%	100%	100%	100%	100%

*p-*value was noted in the table only if there were statistically significant differences between groups.

**Table 9:** General characteristics of respondents’ responses in the VARSEEK construct.

	Average Score	Group Size	% of the Population
Group I (low level of seeking diversity in food)	1.0–2.2	29	6.40%
Group II (medium level of seeking diversity in food)	2.3–3.7	171	37.75%
Group III (high level of seeking diversity in food)	3.8–5.0	253	55.85%

**Table 10:** Agreement of the respondents to the statements of the VARSEEK scale (average values).

Statement	1.	2.	3.	4.	5.	6.	7. (N)	8.
Overall average for the statement	3.32	3.89	4.06	4.23	3.62	3.89	2.67	3.86
Gender								
*p-*value							*p* = 0.0270	
Women	3.26	3.97	4.05	4.20	3.52	3.85	2.72	3.90
Men	3.48	3.72	4.07	4.28	3.85	3.99	2.58	3.82
Age								
*p-*value								
≤20	3.15	3.93	4.13	4.26	3.63	3.91	2.39	3.76
21–40	3.35	3.89	4.05	4.22	3.61	3.89	2.71	3.89
Education								
*p-*value			*p* = 0.0074	*p* = 0.0460				
High school	3.26	3.88	4.07	4.26	3.55	3.86	2.55	3.83
University	3.39	3.92	4.05	4.19	3.69	3.92	2.81	3.92
Financial situation								
*p-*value		*p* = 0.0005				*p* = 0.0070	*p* = 0.0206	
Very good	3.57	3.77	4.15	4.43	3.89	4.03	3.07	4.18
Good	3.29	3.92	4.06	4.24	3.63	3.90	2.62	3.87
Sufficient	3.24	3.90	4.00	4.10	3.43	3.76	2.57	3.70
Bad	3.83	3.67	4.50	4.00	3.67	4.33	3.00	4.00
Subjective evaluation of nutrition								
*p-*value	*p* = 0.00039	*p* = 0.000039	*p* = 0.0000028	*p* = 0.0021	*p* = 0.000403	*p* = 0.0021	*p* = 0.0025	*p* = 0.00019
Very good	4.14	4.50	4.60	4.64	4.26	4.40	3.33	4.36
Good	3.42	4.06	4.19	4.36	3.79	4.02	2.74	4.06
Common	3.07	3.64	3.83	4.05	3.36	3.69	2.52	3.59
Bad	3.21	3.50	3.88	3.83	3.08	3.50	2.25	3.75
Game consumption								
*p-*value	*p* = 0.000637		*p* = 0.0250	*p* = 0.000024	*p* = 0.00023	*p* = 0.000305	*p* = 0.0330	*p* = 0.000255
Yes	3.51	3.94	4.16	4.35	3.80	4.05	2.77	4.00
No	2.94	3.79	3.85	3.96	3.23	3.56	2.48	3.60

*p-*value was noted in the table only if there were statistically significant differences between groups.

**Table 11:** Characteristics of the level of willingness to look for a variety of food in particular groups of respondents.

The Level of Variety Seeking in Food	Gender		Age		Education		Game Consumption		Overall
	Statistical Differences (*p*-value)								
							*p* = 0.0063		
	Women	Men	≤20	21–40	High School	University	Yes	No	
Low	6.01%	7.30%	1.85%	7.02%	8.33%	4.23%	4.87%	9.66%	6.40%
Medium	39.56%	33.58%	48.15%	36.34%	37.50%	38.03%	32.14%	49.66%	37.75%
High	54.43%	59.12%	50.00%	56.64%	54.17%	57.75%	62.99%	40.69%	55.85%
Overall	100%	100%	100%	100%	100%	100%	100%	100%	100%

*p-*value was noted in the table only if there were statistically significant differences between groups.

**Table 12:** General characteristics of the respondents’ responses in the GMAS construct.

	Average Score	Group Size	% of the Population
Group I (negative attitudes)	1.0–2.2	32	7.06%
Group II (ambivalent attitudes)	2.3–3.7	347	76.60%
Group III (positive attitudes)	3.8–5.0	74	16.34%

**Table 13:** Compatibility of the respondents’ opinions with the statements of the GMAS scale (average values).

Statements	1.	2.	3.	4.	5.	6.	7.	8.	9.	10.
Overall average for the statement	2.98	3.58	3.06	3.05	4.02	2.59	2.99	3.39	3.14	2.75
Sex										
*p-*value	*p* = 2.29 × 10^−5^		*p* = 8.9 × 10^−7^	*p* = 2.82 × 10^−5^						
Women	2.81	3.59	2.82	2.84	4.01	2.52	2.94	3.35	3.09	2.75
Men	3.37	3.56	3.59	3.53	4.06	2.77	3.10	3.49	3.26	2.75
Age										
*p-*value										
≤20	3.16	3.43	3.10	3.08	3.90	2.88	2.73	3.37	3.14	2.82
21–40	2.96	3.60	3.05	3.04	4.04	2.56	3.02	3.40	3.14	2.74
Education										
*p-*value						*p* = 0.0160		*p* = 0.0490		
High school	3.02	3.48	3.11	3.13	3.96	2.68	2.90	3.32	3.11	2.75
University	2.94	3.69	2.99	2.96	4.09	2.49	3.08	3.48	3.17	2.74
Financial situation										
*p-*value										
Very good	3.21	3.67	3.10	3.11	4.15	2.67	3.25	3.61	3.33	3.07
Good	2.93	3.58	3.07	3.06	4.01	2.58	2.94	3.39	3.08	2.68
Sufficient	2.98	3.56	3.00	2.98	4.00	2.59	2.96	3.32	3.19	2.75
Bad	2.83	3.00	3.17	3.17	3.83	2.67	3.00	2.83	2.83	2.83
Subjective evaluation of the way of nutrition										
*p-*value	*p* = 0.0113		*p* = 0.0465	*p* = 0.0266		*p* = 0.0078				
Very good	3.17	3.60	2.86	2.74	4.14	2.17	2.83	3.57	3.33	3.12
Good	3.10	3.61	3.21	3.19	4.01	2.68	3.07	3.53	3.25	2.76
Common	2.83	3.57	2.97	2.99	3.97	2.64	2.95	3.25	3.02	2.68
Bad	2.92	3.42	2.83	2.83	4.29	2.29	2.88	3.08	2.96	2.54
Game consumption										
*p-*value	*p* = 1.37 × 10^−12^	*p* = 0.00248	*p* = 9.1 × 10^−15^	*p* = 3.4 × 10^−15^	*p* = 0.0204		*p* = 7.8 × 10^−5^	*p* = 0.00115	*p* = 1.002 × 10^−5^	
Yes	3.27	3.69	3.43	3.43	4.11	2.59	3.16	3.54	3.31	2.77
No	2.37	3.36	2.26	2.25	3.83	2.61	2.62	3.08	2.79	2.70

*p-*value was noted in the table only if there were statistically significant differences between groups.

**Table 14:** Characteristics of attitudes towards game in particular groups of respondents.

Attitudes towards Game Meat	Gender		Age		Education		Game Consumption		Overall
	Statistical Differences (*p*-value)								
	*p* = 0.0170						***p* = 0.00011**		
	Women	Men	≤20	21–40	High School	University	Yes	No	
Negative	9.49%	1.46%	6.12%	7.18%	6.67%	7.51%	3.90%	13.79%	7.06%
Ambivalent	76.90%	75.91%	77.55%	76.49%	77.08%	76.06%	74.03%	82.07%	76.60%
Positive	13.61%	22.63%	16.33%	16.34%	16.25%	16.43%	22.08%	4.14%	16.34%
Overall	100%	100%	100%	100%	100%	100%	100%	100%	100%

*p-*value was noted in the table only if there were statistically significant differences between groups.

## Data Availability

The data presented in this study are available on request from the corresponding author. The data are not publicly available due to privacy.
